# Resistance and Reproduction: Mental Illness Stigma Observed in a Chinese Psychiatric Hospital

**DOI:** 10.1007/s11013-026-09980-5

**Published:** 2026-04-09

**Authors:** Zhuyun Lin

**Affiliations:** University of Hong Kong, Hong Kong, China

**Keywords:** Mental illness, Stigma resistance, Reproduction, Psychiatric hospital

## Abstract

Grounded in stigma resistance theory, this ethnographic study identifies resistance strategies employed by patients and families within a Chinese psychiatric hospital, often in collaboration with institutional practices. While denial manifested as refusal to acknowledge diagnoses, distancing involved both social avoidance of peers and spatial negotiations. These strategies are characterized by subtle and personalized actions that receive limited attention. Although patients and families actively resist stigma, their efforts are constrained by the lack of supportive networks and the entrenched structural stigma within mental health systems, reinforcing the existing power dynamics within the facility. To meaningfully address and dismantle these entrenched structures, it is imperative to foster collaborative frameworks that bridge the perspectives of patients, families, and healthcare providers.

## Introduction

While conducting fieldwork in a Chinese psychiatric hospital in Guangdong province, I observed an intriguing phenomenon: patients’ pervasive denial of diagnoses. Despite repeated admissions to the facility, the majority of patients refused to acknowledge their mental illness, rejecting the identity of being a mental health patient. Upon initial engagement with the nursing and psychiatric staff, including social workers, I was told that this lack of disease recognition was, in fact, a symptomatic feature of the illness itself. This is a generalized way for medical staff to refer to patients in their daily communication, and it is not specifically directed at any particular disease. This raised a critical question: Why did patients resist being associated with mental illness? If acknowledging their condition and adhering to treatment could improve their health outcomes (Kim et al., 2020; Pereira et al., 2024), why were patients reluctant to collaborate with healthcare professionals for a quicker recovery?

Studies show that between 50% and 80% of people diagnosed with schizophrenia have limited or no awareness of their condition (Lincoln et al., 2007; Ouzir et al., 2012). This lack of insight is often linked to lower adherence to treatment, which can lead to worse health outcomes and reduced daily functioning. However, research examining the factors associated with insight and its long-term impact has produced mixed results (Lincoln et al., 2007). This paradoxical finding underscores the complexity of the issue and highlights the need for a deeper understanding of the phenomenon of mental illness denial in a more nuanced way within institutions.

Stigma surrounding mental illness—including public stigma, self-stigma, and structural stigma—remains a major barrier to care and is associated with adverse outcomes (Chukwuma et al., 2024; Eziechi et al., 2025). The labeling process often reinforces feelings of shame and denial, leading individuals to reject or conceal their mental health challenges to avoid prejudice and discrimination (Ben-Zeev et al., 2010; da Silva et al., 2020). In clinical practice, denial is commonly interpreted as a sign of impaired insight (Lincoln et al., 2007; Ouzir et al., 2012). However, is all denial simply a symptom? Or can refusal carry other meanings—such as resistance to the stigma attached to psychiatric labels?

Existing research on stigma resistance has focused primarily on community and outpatient contexts, where strategies such as advocacy and identity management are more visible (Swistak et al., 2024; Thoits, 2011). Studies show that stigma resistance is linked to higher self-esteem and better recovery outcomes (Dubreucq et al., 2022; Lau et al., 2017). Yet, little empirical work has examined how resistance operates within psychiatric hospitals, where institutional structures and routines shape patient experiences. Investigating these dynamics offers critical insight—not only challenging conventional clinical interpretations of denial, but also informing alternative approaches to address stigma within healthcare institutions.

This study addresses this gap by adopting ethnographic fieldwork as a more nuanced approach to examine stigma resistance within a Chinese psychiatric hospital, using Thoits’s stigma resistance theory as an interpretive lens. It explores: (1) What disengagement strategies do patients and families employ in the hospital setting? (2) How do these strategies demonstrate the dynamics of psychiatric stigma within institutional environments? By answering these questions, the study uncovers characteristics and consequences of stigma resistance in inpatient care.

## Stigma Resistance

The study of stigma has evolved since its early conceptualizations, particularly influenced by Erving Goffman’s seminal work (1963), in which he describes stigma as “an attribute that links a person to an undesirable stereotype,” leading to the discrediting, devaluation, and discrimination of those who possess the stigmatized attribute. Goffman’s framework focused on the micro-level interactions and the individual experiences of stigmatized individuals, emphasizing the social and psychological processes through which stigma is internalized and managed in everyday life. While Goffman’s work provided a foundational understanding of stigma, it has been critiqued for its relative neglect of the broader structural and power dynamics that underpin the production and perpetuation of stigma (Link & Phelan, 2001). This oversight limited the early discourse on stigma, as it failed to fully address how stigma operates as a tool of social control, reinforcing hierarchies and suppressing marginalized groups.

In response to this gap, scholars began to shift their focus toward the macro-level dimensions of stigma, examining how it is systematically produced and institutionalized to maintain power imbalances (Sebrechts, 2024). This critical turn highlighted the role of societal institutions, cultural norms, and political structures in creating and sustaining stigma. For instance, Link and Phelan’s (2001) conceptualization of stigma as a process of labeling, stereotyping, and social exclusion underscored the power dynamics inherent in stigmatization. They argued that stigma is not merely an individual experience, but a mechanism through which dominant groups exert control over marginalized populations, such as racial minorities, individuals with mental illness, or those living in poverty. This macro-level perspective brought attention to the systemic nature of stigma, revealing how it is embedded in social, economic, and political systems to perpetuate inequality (Parker & Aggleton, 2007).

More recently, the literature on stigma has undergone another shift, moving beyond the focus on its production and effects to explore the ways in which stigmatized individuals and communities resist and challenge stigma. This emerging body of work emphasizes the agency of marginalized groups and their capacity to engage in acts of resistance, both overt and covert, in their daily lives (Campbell & Deacon, 2006; Jenkins & Carpenter-Song, 2008; Nahar & van der Geest, 2014; Sibitz et al., 2011; Tyler & Slater, 2018; Wahl, 1999). Scholars have begun to document the diverse strategies employed by stigmatized individuals to contest and redefine stigmatized identities, ranging from collective activism to subtle, everyday acts of defiance. For example, research has highlighted how individuals with mental illness use narrative practices to reclaim their identities (Wahl, 1999), or how women in Bangladesh confront the stigma of childlessness and create space for themselves in their daily lives (Nahar & van der Geest, 2014).

This recent emphasis on resistance has also led to a deeper exploration of the “everyday world” of stigmatized individuals, examining how resistance is woven into the fabric of daily life. Rather than viewing resistance as solely large-scale or organized movements, scholars have turned their attention to the micro-level, mundane acts of defiance that occur in routine interactions (Beaupre, 2023; Masaki, 2006). These acts, though often invisible or overlooked, play a crucial role in undermining the power of stigma and asserting the dignity and agency of marginalized groups (Scott, 1990). Through an exploration of everyday resistance in sheltered workshops, Sebrechts observed that such resistance is rooted in diverse desires and needs, manifesting in subtle, unorganized, and implicit ways (Sebrechts, 2024).

Stigma resistance involves the active rejection of societal stereotypes, rather than their internalization, manifesting in diverse emotional and behavioral responses such as anger, indifference, and empowerment (Corrigan & Watson, 2002; Thoits, 2011). Coping strategies in stigma resistance are broadly categorized into engagement—confronting stigma directly, such as challenging—and disengagement, such as deflecting—indirectly rejecting the mental patient identity (Crocker & Major, 1989; Miller & Kaiser, 2001; Thoits, 2011). In this study, I will focus on disengagement. While previous research has highlighted active forms of resistance such as advocacy or public confrontation, these strategies are often constrained in clinical settings, particularly inpatient psychiatric environments, where hierarchical structures and rigid routines limit opportunities for overt opposition (Goffman, 1961; Slemon & Dhari, 2024). In such contexts, disengagement—defined as a psychological and behavioral process of distancing oneself from stigmatizing interactions or institutional practices—becomes a more feasible and adaptive form of resistance. Disengagement allows patients to preserve self-worth and autonomy without directly challenging institutional authority. Therefore, examining disengagement provides a context-sensitive lens for understanding stigma resistance in psychiatric hospitals, where structural and interpersonal stigma intersect with limited patient agency.

In her research, Thoits (2011) describes deflection as a cognitive strategy of resisting stigma, whereby individuals reject, rebut, or refute the notion that they have a mental disorder or that they identify as a “mental patient.” For example, individuals—particularly those with less severe mental illness—often distance themselves from stigmatized groups by accepting public stereotypes as accurate, but refusing to apply these labels to themselves (Thoits, 2016). Thoits (2011) emphasizes that stigma resistance is conceptually distinct from lack of insight. Resistance presupposes awareness: individuals must recognize that the label of “mentally ill” or “mental patient” could potentially apply to them even if they reject them, understand that this label can become a public identity if disclosed, and know the cultural meanings attached to it—even if they reject those meanings (Thoits, 2011). As Thoits (2011) observes—echoing Corrigan and Watson’s (2002) findings—these three preconditions “distinguish individuals who employ resistance and other coping strategies from those who remain unaware of their illness or of cultural stereotypes, often due to the illness itself.” In this context, patients who are defined as resisting the stigma are aware of their illness and its implications, even if they outwardly deny it. For these individuals, what appears to be denial can in fact function as a form of resistance. In contrast, denial rooted in lack of insight reflects unawareness of symptoms or the need for treatment (Lincoln et al., 2007; Ouzir et al., 2012).

Nevertheless, within the facilities, clinical staff often interpret denial as symptomatic of impaired insight; my observations suggest a more nuanced reality: while some denials do indicate lack of insight, others—particularly among patients with mild to moderate symptoms, no prior treatment history, awareness of stigma, and multiple social roles (Thoits, 2011)—represent a conscious rejection of the “mental patient” identity. These patients meet Thoits’ preconditions for resistance, acknowledging the potential applicability of the label, while actively resisting its stigmatizing implications.

Much of the existing literature on stigma deflection strategies focuses on psychiatric patients in community settings, where resistance often centers on rejecting identification as a “mental patient” or distancing from psychiatric symptoms in public (Dubreucq et al., 2022; Thoits, 2016). However, institutional contexts present different dynamics. Within hospital environments, some patients deny their illness because they do not perceive themselves as “mad people”—a category culturally associated with dangerousness and loss of control—thereby distancing themselves from other patients. Others, particularly those who are newly admitted or exhibit milder symptoms, may reject the “mental patient” label as a way to assert distinctiveness during interactions with clinicians or social workers. These observations underscore the importance of examining denial within its specific context and in relation to patients’ perceptions of mental illness stigma. In inpatient settings, denial may not always signify impaired insight; rather, it can function as an everyday form of resistance—specifically, disengagement from stigma—aimed at preserving identity and autonomy (Sum et al., 2024; Thoits, 2011).

Furthermore, much of the previous research on families of people with mental illness has interpreted their delayed help-seeking behavior as a form of stigma avoidance (Shi et al., 2019). However, if we consider the course of the illness over time, it becomes clear that what is often labeled as “avoidance” may in fact be a more nuanced response. Families, acutely aware of the impact of mental illness stigma, may interpret the patient’s symptoms as mild compared to “madness,” and thus resist labeling their loved one as mentally ill until it becomes absolutely necessary. In this sense, their actions can be seen as a form of stigma resistance, shaped by their understanding of the illness at different stages. Although families ultimately accept the legitimacy of institutional care by admitting their relatives, they may also serve as advocates. Through what Grainger (2021) calls “proxy deflection,” families help patients resist the stigma associated with mental illness, even within hospital settings—which is often overlooked. Therefore, this study also examines how families employ proxy deflection strategies within the hospital, how these actions unfold in the clinical context, and what impact they may have.

Engagement and disengagement constitute two distinct forms of stigma resistance, each with their own consequences. Engagement—actively challenging stigma—has the potential to boost self-esteem, even if immediate results are limited (Marcussen et al., 2021), while disengagement serves primarily to protect self-esteem by avoiding stigma-related threats (Corrigan et al., 2016; Rüsch et al., 2005; Sum et al., 2024). These findings highlight the complex and multifaceted nature of stigma resistance, which not only mitigates the harmful effects of stigma, but also fosters psychological resilience and well-being. This study thus will contribute to advancing understanding of stigma resistance strategies within healthcare institutions—a domain that has received limited scholarly attention to date.

## Mental Illness Stigma in China

Existing research underscores that stigma remains a critical barrier to mental health help-seeking in China, compounded by systemic inequities in resource distribution between urban and rural areas, and entrenched sociocultural norms (Hu et al., 2021; X. Xu et al., 2018; Yin et al., 2019). Studies highlight how stigma manifests across diverse social spheres, including workplaces, communities, and families, where mental illness is frequently associated with a loss of “face” (Wong et al., 2018; J. Yang, 2017; L. H. Yang & Kleinman, 2008; Zhang et al., 2020). Families often conceal diagnoses to avoid social judgment, isolating patients and worsening their conditions (Li & Rose, 2017; Ran et al., 2018). Media representations further entrench public stigma by framing psychiatric patients negatively (Zhang et al., 2020). However, in community mental health services, there remains a lack of services in the area of anti-stigma efforts.

In China, the emergence of community mental health initiatives can be traced to the 2004 launch of the “686 Project,” so named for its initial allocation of 6.86 million RMB. Conceived as an effort to integrate hospital-based and community mental health services (Liang et al., 2018; H. Ma, 2012), the project nevertheless fell short of its aspirational goals. In practice, community mental health services have often operated less as vehicles of care than as instruments of biopolitical regulation. As Ma (2020) and Zhu et al. (2018) observe, such programs tend to frame individuals with mental illness primarily as subjects of risk, privileging control over therapeutic engagement. This orientation has contributed to the entrenchment of stigma within community settings, and rendered family caregiving increasingly precarious. Institutional structures, in this sense, not only reflect but also reinforce the marginalization of people living with mental illness, and interventions aimed at destigmatization have produced only modest effects. The consequences of these dynamics are evident in the everyday experiences of service users. Lin’s (2025) study offers a striking example through Qiong, an outpatient who persistently sought to live what she considered a “normal” life within her rural community. Despite her efforts—working, maintaining family roles, and engaging socially—she remained marginalized by villagers who continued to view her through the lens of mental illness stigma. Lin’s analysis demonstrates how entrenched cultural perceptions and moral judgments can override individual attempts at reintegration, revealing stigma as a pervasive social and moral force that shapes identity and belonging (Lin, 2025).

### Local History of Institutional Care

Mental health care in Southern China has long reflected the negotiation between local cultural practices and biomedical paradigms. Before 1949, philanthropic organizations and missionary hospitals laid the foundation for institutional care, and by the mid-twentieth century, custodial asylums had emerged, though therapeutic treatment remained limited until the establishment of “F Hospital” in 1969. Despite these developments, understandings of mental illness continued to draw on traditional Chinese medicine (TCM) concepts such as *kuang* (狂) and *dian* (癫), and families typically sought TCM or folk remedies before turning to psychiatric care—a pattern shaped by stigma and cultural norms (Baum, 2018; Lin & Ma, 2023). Medical records from the 1970s show that patients often presented with somatic complaints like headaches and insomnia, reflecting culturally sanctioned idioms for distress and strategies to avoid psychiatric labeling (Baum & Lin, 2022; Kleinman, 1988; Lee, 1999). Even in recent years, families frequently attribute symptoms to spiritual causes, seeking ritual interventions before reluctantly engaging biomedical services (Lin & Ma, 2023). These practices underscore the persistence of local explanatory models and the enduring stigma surrounding psychiatric treatment.

My fieldwork at F Hospital suggests that this stigma is not only pervasive, but often treated as self-evident in everyday life. Friends expressed fear about my work in the psychiatric hospital, describing it as a place to avoid, and conversations about mental illness were marked by discomfort and silence. Healthcare workers acknowledged stigma but framed it as a problem external to the hospital, rarely recognizing its presence within institutional routines. Meanwhile, patients and families were acutely aware of discrimination in the community, viewing it as an unquestioned reality. These observations highlight the need to examine how stigma is locally perceived, discussed, and negotiated—not only in community settings, but also within hospitals, where its effects are often overlooked. Grounded in the aforementioned literature, and despite mental health policy shifts toward community-based care, institutional care persists as the dominant model, inadvertently reinforcing stigma through hospital-centric practices (Han et al., 2020; Xu et al., 2018). However, few studies have explored stigma resistance in China, particularly within psychiatric hospitals, where power dynamics between service providers, patients, and families may reshape our understandings of how patients and families navigate and resist stigma during medical encounters.

## Methodology

In this study, I have employed an ethnographic fieldwork approach, while relying primarily on the analysis of service records generated in my previous working experience. Although ethnography traditionally involves immersive, in-person field engagement, its analytical orientation is equally valuable for interpreting documentary materials produced within sensitive contexts such as mental health settings (Balcom et al., 2021; Browne & McBride, 2015). By reading service records ethnographically, the study attends to the lived experiences, social interactions, and subtle institutional dynamics embedded in everyday documentation—insights that may remain obscured in interview-based research alone. This approach provides a nuanced understanding of how stigma and resistance are articulated, negotiated, and recorded in daily clinical practice.

### Statement of Ethics

Prior to the commencement of the study, the research protocol was reviewed and approved by the Ethics Committee at F Hospital, which is composed of specialists from various medical departments. All procedures were designed and conducted in accordance with the ethical standards set forth in the 1964 Declaration of Helsinki and its subsequent amendments. Participation in interviews with medical staff was voluntary; participants were informed of their right to withdraw from the study at any time without consequence. To ensure confidentiality and privacy, pseudonyms were assigned to all participants and any identifying information was removed from the data. For patient and family data, only anonymized service records were used with institutional approval, and no direct research interviews were conducted with them in this study.

### F Hospital

The data presented in this article originate from a 16-month fieldwork study from a period between 2017 and 2019 at F Hospital, a city-level psychiatric hospital with 1,000 beds and 500 medical workers. Situated in a suburban area, the medical space at F Hospital is constituted mainly of the psychiatry department buildings, which form a closed area for patients admitted to the department. There are subdivisions within the department, which is made up of five wards (numbered second to sixth). Patients brought in by family members usually present with severe symptoms. Most are first assessed by psychiatric physicians in the outpatient clinic and then admitted directly to the emergency room of the appropriate ward for observation. In cases of acute psychotic episodes, some patients are sent straight to the emergency room without any prior outpatient evaluation. These patients are subsequently assigned to the second through fifth wards, located in a four-story building. I refer to these as “general wards,” which together house approximately 400 patients. Each of these wards has its own team of doctors who specialize in different types of mental disorders. For example, the fifth ward primarily admits female patients with bipolar disorder or other affective disorders. In some cases, if a patient’s family has connections with particular medical staff, the patient may be admitted to a specific ward based on those relationships.

Historically, psychiatry departments initially accommodated both male and female patients together. However, as patient numbers increased, administrators found that mixed-gender wards were difficult to manage, particularly due to concerns about inappropriate sexual interactions between male and female patients. As a result, hospitals instituted gender segregation, assigning male and female patients to separate wards. Divided by gender, the second and third wards are male wards, and the fourth and fifth wards are female wards (the former “first ward” has been renamed Geriatric Ward and the sixth ward is for homeless patients).

### Data Collection

Data for this study were drawn from multiple sources, including fieldwork notes, service records, and interviews with medical staff. Interviews with healthcare workers were primarily conducted in 2018 and 2019, and arranged through personal introductions and occasional informal encounters. I engaged with most patients and their families during service provision in 2016; information about these participants comes mainly from service records—which include background details (see Table 1)—and field notes documenting institutional interactional contexts in 2019. Information on healthcare professionals comes from both field notes and interviews, which explored their work experiences, the history of the hospital’s development, and the evolution of treatment methods over time. The sample consisted of 23 medical staff members, including 7 doctors, 9 nurses, 3 pharmacists, 2 social workers, 1 administrative staff member, and 1 technician. Participants ranged in age from 22 to 86 years, with a mix of employed and retired professionals (see Table 2). To protect privacy, all names mentioned in this article have been anonymized.

**Table 1:** Patients’ background information

Participant	Gender	Age	Diagnosis	Course of illness (by year)
Patient Min	Female	48	Schizophrenia	10
Patient Hui	Male	23	Schizophrenia	8
Patient Nana	Female	20	Schizophrenia	2
Patient Shu	Female	14	Adolescent affective disorder	1

**Table 2:** Background information on the medical staff

Role	Count	Gender (M/F)	Age range	Employment status when interviewed
Doctors	7	6M/1F	25–86	Mostly employed; some retired
Nurses	9	0M/9F	22–75	Mixed employed and retired
Pharmacists	3	2M/1F	58–86	Mixed employed and retired
Social Workers	2	0M/2F	32–45	Employed
Admin Staff	1	0M/1F	30	Employed
Technicians	1	0M/1F	22	Employed

Although the hospital was cautious about patients’ contact with outsiders, my role as a social worker intern alleviated such concerns, allowing me to understand their mental illness experiences in a more organic way. I would contact patients and family members under the role of social worker. Some I had worked with during the service process, and data for these patients come primarily from service records. This included information such as the initial reasons for their hospitalizations and their life experiences with mental illness outside the hospital. For family members, the service record showed how they had recognized the patients’ “abnormal” behavior, the admission process, and how they had coped with the patients and managed their lives after discharge. I conducted case services and organized activities for the patients within the facility. By participating in their daily activities at the hospital, I was able to record some essential details, such as participants’ interactions and their responses to the medical arrangement.

The interviews I conducted with medical workers were semi-structured, and participants were informed about the research and its purpose. Interviews with medical workers began by inquiring about their motivation to become mental health workers and their reasons for choosing to work in a psychiatric hospital. They were also given the opportunity to share their experiences with patients and the associated stigma they had encountered in their daily lives. Most of the interviews took place within the hospital and most of the records had been produced within the hospital. The interviews lasted between half an hour and one hour and were mostly conducted in Mandarin Chinese. The data related to the research have been anonymized and are securely stored in password-protected and encrypted computers and external hard disks.

### Statement of My Positionality in the Field

My fieldwork at F Hospital was shaped by my multiple, overlapping roles. I had initially gained access to this patient population as a social worker in 2016. Upon my return in 2019 as both an intern and a researcher, many patients still saw me as a service provider. To gain access to historical archives and service records, I leveraged my existing staff connections to secure internal research projects. This positioned me as a partial insider: familiar with the hospital's workings yet not part of its inner circles. This ambiguity required constant negotiation of my identity. My gender facilitated rapport, particularly with female patients who often saw me as an ally, though my youth and affiliation with the medical staff marked me as an outsider to their lived experiences. Colleagues even advised concealing parts of my role to avoid distressing patients. Navigating these identities heightened my awareness of the ethical boundaries between social work and research. I practiced reflexivity, deliberately separating my service duties from my research aims to avoid exploiting my position. By analyzing service records rather than direct interactions, I minimized disruption to care, while enabling critical analysis of my observations.

### Data Analysis

The recorded interviews were transcribed in both Chinese and English for analysis. After transcription, each transcript was carefully checked for accuracy and completeness, including verification of audio-text alignment, removal of any typographical errors, and consistency in translation between Chinese and English. Following this quality check, I coded and analyzed the transcripts using the thematic analysis approach (Braun & Clarke, 2021). Based on the findings from interviews and participant observations, I attempted to identify the categories of stigma resistance strategies and observe how stigma was reproduced in daily medical practice.

## Results

My ethnographic fieldwork documented various disengagement strategies patients and their families employed within the psychiatric hospital, alongside the institution’s tacit collaboration in these efforts. The following section details these strategies and their implications for understanding stigma as a dynamic, institutionally embedded process.

### I am not Someone Like Them

Within the hospital, patients often engaged in comparison with one another, actively distinguishing themselves from those they perceived as having more severe psychiatric conditions. Patients frequently practiced conscious denial of their own diagnoses. For instance, Hui, a 25-year-old man diagnosed with schizophrenia and who had been hospitalized three times, insisted to his social worker, “The other patients are mentally ill, not me,” and expressed an aversion to associating with other psychiatric patients. Hui was cognizant of the stigma and discrimination attached to mental illness—he knew that after his diagnosis, his sister had stopped contacting him, and his circle of friends had diminished. Nevertheless, he did not perceive himself as the kind of “mentally ill” person others imagined. As a result, Hui often kept to himself in the ward and intentionally distanced himself from other patients. He felt that his own behavior was much better than that of those in the acute ward or those who were incoherent or unable to control themselves, and that he did not resemble them. Although he had argued with his mother about being discharged—his mother had insisted he was ill and needed treatment, while he had attributed his problems more to family issues than to illness—Hui continued to reject the psychiatric label. On one occasion, he volunteered to participate in my group activity; when I asked him why, he said he had some issues with communication and behavior. However, during the activity, he suddenly remarked that he “is a mental patient,” though, overall, he continued to assert his difference from others. By distancing himself from those he perceived as more severely ill, Hui rejected the stigmatized identity associated with being a psychiatric patient.

Similarly, Xia, a middle-aged woman diagnosed with bipolar disorder, also maintained a sense of distinction between herself and other patients. She attributed her presence in the psychiatric ward to her noncompliance with medical advice, rather than to being “mad” like the others. When I first met her, she had just been transferred from the clinical psychology department to the psychiatric department and was helping another female patient analyze her problems. Xia insisted that her thinking was rational and clear, unlike the “irrational” or “out of control” behavior she had observed in others.

These cases demonstrate how patients employ distancing strategies—such as diagnostic denial and social distancing of peers exhibiting overt symptoms—as protective mechanisms against the internalization of stigma. In addition to verbal disclaimers, some patients expressed their rejection of the psychiatric label through daily actions. For example, Min, a middle-aged woman, was skeptical of her doctor’s diagnosis, insisting that she was only admitted to the hospital due to depression, and has remained there simply because her family has not come to take her home. She maintained daily routines of reading and exercise to counteract the side-effects of medication, further reinforcing her sense of difference from other patients:

Prior to being admitted to the hospital, I had worked in a music production company, with a [monthly] salary of 1,300 RMB. I knew a man there but we broke up. My job depressed me and together with my emotional affairs, my sister said that I was a little mad during that period. So I visited F Hospital and was diagnosed with depression.

I got married at 30 years old. But I divorced after giving birth. I didn’t like that man. He insisted on marrying me even though I was suffering from depression at that time. After I was admitted, he visited me once, later he didn’t show up. I would not blame him. He was not a rich man, and he had a family to support.

The doctors who were responsible for me changed quite often. Initially, they gave me the diagnosis of depression, then schizophrenia. Once a patient sleeping beside my bed said I was a “hooker”. I was angry and covered her mouth; then the doctor said I had mania, then they increased the dosage of my medication and punished me with electronic shocks. Doctor Ou, my former doctor, I remembered that time she was pregnant. She said I was a normal person. You see, who doesn’t have a temper? They required me to control my temper, telling me that whatever other patients said, I should take it quietly.

Taking psychotic drugs easily causes obesity. They have side-effects. You see my hand is shaking. Many patients take medicine, and there is nothing to do here. They eat and sleep, sleep and eat. Even though the meal is unappetizing, if you put medicine in it, you will easily get sleepy after taking it. Many patients have obesity issues. I used to dance and I was in good shape. Now, to resist the effects of drugs, I do sit-ups every day, 200 of them before going to bed. The nurses don’t believe me that I do it” (Service record, 2016/4/1).

Min demonstrated considerable knowledge about her own condition, having navigated changes in her diagnosis from her initial experience with depression to subsequent encounters within the hospital. While she may have accepted her diagnosis of depression, she resisted any suggestion of conditions such as schizophrenia. Despite these shifts, she consistently insisted that she was a “normal person,” strategically “borrowing” the words of a doctor to articulate her rejection of labels associated with severe mental illness. To further assert her normalcy, Min pointed to her disciplined routines in the hospital—for example, maintaining a regular exercise regimen and continuing to see herself as a woman who manages her figure well. During a follow-up conversation in 2019, Min also revealed her awareness of stigma in the community, acknowledging that discrimination from villagers is a real concern after discharge. Through these strategies, Min actively resists the stigma of mental illness, both within the hospital and in anticipation of community stigma.

### They are not That Mad

It is not only the patients themselves who enact strategies to reject the label of mental illness within the hospital context. If closely examining the process by which family members seek care for patients, it becomes evident that families often practice a form of proxy resistance to the psychiatric label, particularly in the early stages of symptom onset. For patients who are newly experiencing mental illness—especially young people—their families often act as advocates on their behalf. In the early stages of illness, families tend to hold onto hope, believing that their loved one’s condition is not serious, and is fundamentally different from those who are considered “mad.” This resistance often manifests in a reluctance to label their relatives’ condition as a severe mental illness. Instead, they prefer to interpret the symptoms as mild or temporary, seek placement in departments with less stigmatizing names, reject resources that might carry stigma, and sometimes even request a change in diagnosis. These strategies reflect the family’s acute awareness of the stigma attached to mental illness and wanting to reject the identity of mental patient for their love ones.

#### Rejecting Resources Attached to Severe Mental Illness

The decision to avoid psychiatric care until Nana’s condition had become unmanageable reflects her family’s understanding of mental illness stigma and their attempts to distance themselves from it. At 18, Nana, a high school student, had experienced her first psychotic episodes. After a particularly frightening incident in which she threw a knife at her mother, her family rushed her to the hospital for her first psychiatric admission. Overwhelmed and disoriented by the sudden upheaval, Nana struggled to grasp the reality of her situation, while her mother—deeply shaken—insisted on immediate evaluation. To help Nana stabilize emotionally during her hospitalization, her mother sought support from a social worker, who provided regular guidance and reassurance.

When contextualizing Nana’s situation for the social worker, her mother recounted events leading up to the admission: a year earlier, Nana’s teacher had reported her for stealing money, and had noted her increasing social withdrawal and strained peer relationships. Nana interpreted these experiences as ostracism, a perception corroborated by her teacher. Later, Nana was found wandering her school dormitory in a disoriented state, claiming she was “responding to a summons,” prompting the teacher to recommend a temporary leave from school. At home, Nana’s behavior became increasingly erratic, with signs of paranoia and social avoidance.

Recognizing her daughter’s psychological distress, Nana’s mother initially sought help from psychologists, though Nana rejected these interventions. Despite being aware of psychiatric options, her mother deliberately avoided pursuing a formal mental health diagnosis, fearing the stigma attached to such a label and its potential impact on Nana’s future. For over a year, she prioritized non-medical coping strategies, such as seeking help from local temples and performing ritualistic ceremonies to ward off “bad influences,” during which time Nana’s symptoms progressively worsened. The turning point came when a nurse relative, alarmed by Nana’s deteriorating condition, advocated for psychiatric hospitalization.

Nana’s mother’s initial reluctance to label her daughter with a psychiatric diagnosis stemmed from an awareness of the stigma effect. She preferred to interpret Nana’s early symptoms as mild or remediable through rest and alternative practices, actively distancing her daughter’s condition from the domain of mental illness. As Nana’s caregiver, her mother—like many others—sought to protect her child from the further harm of mental illness stigma. Only after accepting her daughter’s diagnosis did she begin to read relevant literature and share Nana’s experiences with friends and family, expressing hope that Nana could return to a normal life through consistent medication. Even then, she tended to describe Nana’s behavior as “slow” rather than “mad,” resisting the more stigmatized language often associated with mental illness.

A similar dynamic is observed in Hui’s family. Hui’s mother had accepted her son’s psychiatric diagnosis but maintained that he was different from others, for instance, more severely affected patients who were physically incapacitated or overtly psychotic. She carefully planned Hui’s post-discharge life and refused to apply for a disability certificate, not merely because the financial compensation was minimal, but because she believed that acquiring the label of “mentally disabled” would prevent her son from integrating into the community. She continued to hold hope that Hui could marry and establish a family, and through her resistance to disability-related labels, sought to preserve his dignity and shield him from the consequences of stigma:

I wouldn’t apply for this assessment. If he becomes mentally disabled, he will have no chance to live in the community; I still want him to marry and have a family. (Fieldnote, 2019/09/28).

This reluctance to engage with disability-related policies is common among families, as reflected in the declining number of applications for mental disability certification—a trend noted by the nurse responsible for processing these requests. At a mental health system internal working meeting on severe mental illness, officials from the Disabled Persons’ Federation highlighted the persistent challenge of low application rates for mental disability assessments, attributing it to the pervasive stigma surrounding mental illness, which deters families from seeking government support.

In Hui’s case, his mother had weighed the benefits of applying for the certification against the lasting impact of the “mentally disabled” label. For younger patients, families often retain hopes for recovery and future reintegration. For relatives of first-time inpatients, strategies to help patients distance themselves from the mental illness label are crucial to their adaptation to life after discharge. Just as Hui’s mother had refused to apply for psychiatric disability status, Shu’s mother negotiated with doctors to assign her daughter a milder diagnosis, hoping that by distancing her from the severe mental illness label, she could facilitate Shu’s reintegration into the community.

#### Changing Diagnoses

Another strategy families employ to resist stigma involves pressuring clinicians to modify psychiatric diagnoses into less stigmatizing terms. This diagnostic rebranding is intended to distance patients from overtly “mental illness” classifications, while they still access hospital care. By negotiating diagnostic language, families attempt to shield loved ones from societal prejudice, while navigating the contradictory demands of securing treatment and preserving social standing in the community.

Shu’s mother approached the situation with a specific strategy in mind. As Shu’s discharge date approached, her mother knew the impact of stigma in the community. She wanted to secure her daughter’s future integration into the community through normal daily life, and urged the doctor to diagnose Shu with “adolescent affective disorder,” emphasizing that Shu was only a middle school student. However, her mother was unaware that since November 2019 the outpatient reporting system had been conducting automatic risk evaluation for each patient, and had shared this information with the community. According to a 2016 government document titled “Notice on the Implementation Plan for Strengthening the Treatment and Rescue of Patients with Severe Mental Disorders in Xinkang City,” clinical doctors are required to report any individuals with severe mental illness to the Comprehensive Governance Office (CGO) upon discharge. A community guardianship team, consisting of community psychiatric doctors, neighborhood cadres, security cadres, and family members (the guardian), would then be established.

Although Shu had been diagnosed with “adolescent affective disorder,” her doctor had assigned her a risk evaluation of “3.” As per the document, any risk evaluation of “3” or higher had to be reported to the CGO, the neighborhood committee, and the public security department. Consequently, before Shu even returned to her community, her family was unexpectedly “visited” by multiple officials. These sudden visits alarmed Shu’s family, leaving her mother puzzled as to why a diagnosis of “adolescent affective disorder” had triggered such widespread concern. Following these visits, word of Shu’s mental illness spread throughout the community, and her school was also notified. Now, her condition was widely known, which was precisely what her mother had hoped to avoid. To make matters worse, the diagnosis of “adolescent affective disorder,” chosen to resist stigma, was not covered by medical insurance. As a result, her family had to pay around 1,000 RMB per month for Shu’s medication. Had the diagnosis been “schizophrenia,” the medication costs would have been reimbursed. Given the family’s finances, Shu’s mother emphasized that this was a significant expense.

Shu’s mother was frustrated by the unexpected attention and approached the social worker for an explanation of what had happened. Shu’s doctor informed her that the risk evaluation of “3” had been assigned based on Shu’s admission history—she had been brought in by the police twice and had exhibited symptoms such as delusions, including the belief that her mother was not her real mother. The doctor explained that the level 3 rating had been given to ensure her mother’s safety. The social worker thus indicated that patients who were had been brought in by the police, especially those with a history of violence, are typically assigned a level “3” risk evaluation, which is why Shu’s doctor had given her this rating.

Shu’s mother was dissatisfied with the outcome. The hospital social worker then negotiated with the community psychiatric doctor to reduce the frequency of home visits by community staff to Shu’s residence, limiting them to once a week. To explore potential solutions, I consulted Doctor Li, who was responsible for outpatient services, asking if it would be possible to change Shu’s diagnosis to schizophrenia so that insurance would cover her medication costs. Doctor Li said:

The psychiatric diagnosis is scientific and precise; we cannot change it at will. This girl is young. If we give her the diagnosis of “schizophrenia”, she will carry it for the rest of her life. Confronting adolescent cases, doctors will not give them the diagnosis of severe mental illness. Their status will improve and maybe after two or three years, it will change. (Fieldnote, 2019/12/06).

Clinically, adolescent affective disorder and schizophrenia are two distinct psychiatric conditions. However, during adolescence, there can often be symptom overlap and diagnostic ambiguity between affective disorders and early-onset schizophrenia, as adolescent presentations are frequently atypical, and the boundaries between the two are sometimes blurred in the early stages. This clinical overlap may be one of the reasons why Shu’s attending physician was able to adjust the diagnosis in response to her mother’s request. Nevertheless, Shu’s doctor did not share the same priority as her mother—mitigating stigma through a less stigmatizing diagnosis. In other words, the stigma resistance strategy Shu’s mother had employed had been an “under-the-table” effort and had not worked with her doctor. This lack of consensus, combined with the doctor’s risk assessment, in some ways, had perpetuated the stigma of mental illness within the community.

As she had been assigned a level “3” risk evaluation, the community classified Shu as a patient with severe mental illness, placing her under the surveillance and risk management of the community rehabilitation system. Due to teachers’ fear of “risk,” Shu had been withdrawn from school and told to remain at home. During her follow-up visits to the doctor with her mother, I saw Shu several times—a shy, young girl who had been born during her mother’s first marriage. She had been left in the care of her grandparents until her mother remarried and brought her into the new family in the city of Xinkang. This complex family history added another layer to the challenges Shu and her mother faced as they navigated the intersection of mental health care, societal stigma, and personal circumstances.

#### Distinguishing Spatial Arrangement

Within hospital systems, another form of stigma resistance emerges through collaborative distancing between medical staff and families, often materializing in the strategic selection of specialized departments. For instance, the establishment of new departments, such as a clinical psychology department, rather than traditional psychiatric divisions, reflects a negotiated effort to decouple care from stigmatized mental illness classifications. This institutional rebranding, observed in the evolution of hospital departments, allows families to frame hospitalization as treatment for “neurological health” or “stress management,” thereby accessing necessary services while sidestepping the cultural baggage of explicit psychiatric labels.

The psychiatry department is the oldest department in the hospital, established long before the neurology department in the 1980s and the clinical psychology department in the 2000s, which were established to address the evolving needs of mental health care in China, together with the expansion of psy-science (Hsuan-Ying, 2018). The conditions across these three departments vary. The psychiatry department operates under a closed ward system, while the neurology and clinical psychology departments function in an open ward environment. My colleagues told me that the newer departments, which had moved to a spacious modern building, offer better services and equipment, whereas the traditional psychiatry department remains overcrowded and often has an unpleasant odor.

When I first started working at the hospital, an experienced nurse shared a simple way to distinguish the three departments: psychiatry deals with brain-related issues, neurology focuses on neurasthenia (weakness of the nerves), and clinical psychology addresses heart-related concerns. This classification seems to resonate with the understanding of the local slang term “*so*^*4*^,” which means “idiot” in Cantonese. For instance, one time outside my building I observed a conversation between Jia, a retired medical worker who had returned to the hospital, and a group of patients’ family members. The visitors were unsure whether their patient had been admitted to the psychiatry or the neurology department. Jia explained, “These two are different. Neurology is for neurasthenia, and psychiatry is related to “*so*^*4*^.” As he spoke, he pointed to his head, and the visitors quickly understood, responding that the patient had “*nou*^*5*^* so*^*4*^” (a problem with the brain). Jia confirmed that it was the psychiatry department and directed them accordingly.

Similar understanding was shared by another medical staff member who could also speak Cantonese when we discussed a meeting between patients and physicians in Mandarin. The staff member commented:

There is no use in talking to those idiots (*sha zi*). (She paused for a few seconds). No, those living in the neurology department are not idiots, nor are those admitted to the clinical psychology department. They are poor people with some problems in their hearts, like anxiety or depression. But those who live in the psychiatry department are idiots. (Fieldnote, 2019/06/25).

The use of local language, such as the Mandarin term “*sha zi*” or the Cantonese term “*so*^*4*^,” to describe patients in the psychiatry department carried inherently derogatory connotations, reflecting and reinforcing societal prejudices against individuals with mental illness within the facility. Herein, the unconscious adoption of local language within the hospital setting, combined with the spatial and departmental segregation of patients, transforms stigma into a structural phenomenon. However, the initial establishment story provided another view of this differentiation:

The clinical psychology department had a good reputation; indeed, it was the same with the psychiatry department upon its initial establishment. But patients and their family members would accept it if mental illness was called a psychological problem. The expenditure was different: the inpatient fee for one day was 500 yuan in the clinical psychology department (10,000 yuan/month), but only 150 yuan in the psychiatry department. (Fieldnote, 2018/01/26).

Those with desirable status demanded better inpatient living conditions, calling for an independent space and freedom on the ward. (Fieldnote, 2019/11/05).

The establishment of the clinical psychology department had been informed, in part, by families’ concerns about the stigma associated with severe mental illness and their desire to distinguish their relatives from those with more severe psychiatric diagnoses. This consideration aligned with the hospital’s broader strategy for developing clinical psychology as a department characterized by enhanced services and higher fees—costs that placed these services beyond the reach of most families. As a result, the clinical psychology department became a space that attracted patients from higher-income backgrounds, effectively differentiating them from other patient populations. At the same time, this organizational arrangement provided a distinct social and therapeutic milieu for patients and families seeking to resist the stigma of mental illness and assert their difference from those with more severe conditions. However, this very attempt to resist stigma inadvertently reinforced and institutionalized inequality based on economic status within the hospital system; higher-income patients were systematically differentiated from those stigmatized as “idiotic” or “mad” in the psychiatry department, while the better-resourced clinical psychology department became a symbol of privilege, sparking envy among psychiatric patients. Thus, stigma was not merely interpersonal but structural, amplified by the intersection of socioeconomic marginalization and the unconscious use of stigmatizing local language.

## Discussion

This study offers an important contribution to understanding stigma resistance within clinical settings—a domain that has received limited scholarly attention. Existing research on stigma resistance largely focuses on community contexts (Sebrechts, 2024; Thoits, 2011, 2016). In contrast, hospital environments present unique constraints: hierarchical structures, rigid routines, and institutional norms often restrict overt forms of resistance. As a result, strategies in these settings tend to emerge through subtle, everyday practices that are personalized, uncoordinated, and easily overlooked. Situated within the hospital context, this study illuminates how patients and their families employ deflection strategies—including cognitive denial and behavioral distancing—to resist the stigma and social consequences of mental illness. It further explores how healthcare providers respond to these strategies and how such responses shape clinical arrangements and institutional environments, a topic rarely examined in existing literature. By foregrounding these dynamics, this research offers a more nuanced understanding of stigma resistance as it unfolds in inpatient settings, demonstrating that resistance in such contexts does not necessarily reduce stigma, but can produce unintended consequences that reinforce institutional boundaries and social hierarchies.

### Patients’ Denial as a Strategy

Thoits and colleagues’ work on deflection demonstrates that this strategy involves blocking the application of a mental illness identity to oneself, with identity defined by one’s social roles and attributes (Thoits, 2011, 2016). Thus, patients’ denial of illness is influenced by their illness history, symptom severity, awareness of stigma, and social roles. For instance, younger patients experiencing their first episode are more likely to reject psychiatric labels, perceiving themselves as fundamentally different from those who are “truly mad.” This aligns with Thoits’ (2016) study showing that individuals with milder conditions often deny or resist diagnosis, distancing themselves from the more stigmatized identities associated with mental illness. Cases illustrate this: Hui acknowledges interpersonal strain but reframes his difficulties as “a matter of expression”—particularly within family relations—while Min attributes her distress to depression rather than a psychotic disorder. Both explicitly differentiate themselves from “mad people,” indicating awareness of stigma’s social consequences, and they also deny the applicability of the more stigmatized label on themselves.

Within the ward, the salience of stigma is intensified by constant proximity to reference groups—other patients whose severe symptoms are highly visible and become points of comparison. In response, patients like Hui and Min often enact distance as the behavioral component of deflection: limiting contact with other patients, avoiding group activities, and selectively engaging with staff to preserve a non‑patient identity. In this framing, denial (cognitive) and distance (behavioral) are components of one process—deflection—and together constitute a disengagement‑based resistance strategy suited to hospital constraints where overt advocacy is rarely feasible. Yet such “not engaging” behavior is frequently read through a biomedical lens as negative symptoms, for instance social withdrawal, and thus dismissed as mere denial, with little attention to the patient’s experience of stigma or to shifts in stigma perception across the care trajectory. This medicalization—interpreting sociopolitical resistance as clinical symptomatology—reproduces what Kirmayer and colleagues term epistemic violence, erasing the meanings of patient resistance and obscuring systemic inequities (Conrad, 2007; Kirmayer et al., 2014; Scheff, 2017).

### Family Strategies and Institutional Responses

Families, often acting as advocates, may recognize the far-reaching impact of a psychiatric label even more acutely than patients themselves. In efforts to protect their relatives, families adopt what is often characterized in the literature as “avoidance” strategies (L. H. Yang & Kleinman, 2008)—such as secrecy or limiting social interactions to protect the family’s face. However, my findings suggest that this characterization can obscure families’ careful, context-specific calculations. Especially in the early stages of illness, families may use alternative explanations for symptoms, not only due to limited mental health literacy, but also out of a deliberate desire to prevent their loved ones from being branded with a stigmatizing identity. For instance, Nana’s mother initially explained her daughter’s behavior as personality quirks or temporary setbacks, resorting to psychiatric explanations only when symptoms became severe and undeniable. Even then, she sought help and resources that would minimize stigma and foster hope for recovery.

These actions, while appearing to be “avoidance,” are better understood as proxy deflection strategies—families strategically distancing their relatives from the stigma of mental illness while actively seeking support (Grainger, 2021). Unlike mere avoidance, these strategies involve engagement with healthcare resources and a pragmatic acceptance of the diagnosis, coupled with a rejection of the social consequences of the mental illness label, which may lead to withdrawal of the community life. This nuanced resistance is particularly evident among families of hospitalized patients, who may accept psychiatric treatment but resist the stigma, holding on to hope for reintegration and normalcy.

However, family resistance is often unilateral and might not be supported by the healthcare system, and lacks clear cooperation from the patients. For example, Shu’s mother’s efforts to secure a less stigmatizing diagnosis were unsuccessful due to her lack of familiarity with new risk assessment protocols, resulting in intensified stigma and a lack of coping strategies in the community. While some families can partially achieve their goals through negotiation and resourcefulness, such resistance requires coordination and is subject to the discretion and priorities of medical staff.

Another form of resistance involves the strategic selection of hospital departments. The emergence of clinical psychology as a distinct department reflects efforts by some patients and families—often those with greater socioeconomic resources—to distance themselves from the stigma attached to traditional psychiatric patients. This spatial differentiation is both a social and a material process, allowing privileged patients to access less stigmatizing care, while reinforcing hierarchies within the hospital. For example, clinicians may re-label conditions as “neurasthenia” or “depression” to accommodate family preferences and reduce stigma (Kleinman, 1988; Lee, 1999). While such strategies may benefit individual patients, they simultaneously entrench disparities and create new hierarchies of illness, further marginalizing those relegated to more stigmatized psychiatric wards. The differentiation between clinical psychology and psychiatry departments, for instance, not only reflects but also reproduces social inequalities, codifying a hierarchy of mental health care that is mirrored in the language, space, and culture of the hospital. These practices highlight the paradox of stigma resistance within institutions: while intended to protect individuals, they can inadvertently reinforce systemic inequities and perpetuate the very stigmas they seek to reject.

### Limitations of the Study

The findings on stigma presented in this study emerged primarily from fieldwork observations rather than systematic, in-depth interviews with patients or their families. Although I attempted to understand patients’ and families’ perceptions of mental illness stigma during interactions, comprehensive interviews were lacking. While observational data and institutional documents provide valuable insight into patterns of denial and distancing, they cannot fully capture participants’ subjective interpretations—such as whether these behaviors were consciously enacted as stigma resistance strategies to protect or enhance self-esteem (Thoits, 2011). Future research should incorporate qualitative interviews to deepen patients’ subjective understanding of these dynamics and to explore patients’ own accounts of resistance, providing a more comprehensive understanding of the motivations and meanings behind these actions. Furthermore, the reproduction of stigma within the hospital—particularly in relation to local bodily understandings and its association with specific languages—remains underexplored. This research offers a preliminary account based on the available field data. Future studies employing more focused methodologies are needed to deepen and refine the insight presented in this study.

## Conclusion

While patients and families demonstrate significant agency in resisting stigma through everyday acts of distancing and negotiation, their efforts are constrained by the pervasive influence of structural stigma embedded within healthcare systems. The prevailing biomedical model, focused on risk management and clinical symptomatology, overlooks the cultural and political dimensions of stigma (Hinshaw, 2006; Z. Ma, 2020), rendering individual acts of resistance insufficient to challenge systemic inequities (Corrigan et al., 2004).

Despite their best efforts, patients and families often find their resistance co-opted or neutralized by institutional power. As Wacquant and Bourdieu (1992) observe, dissent within such structures is frequently absorbed and transformed into mechanisms of control. Therefore, to effectively address psychiatric stigma, it is essential to foster collaboration among patients, families, and healthcare professionals, and to build supportive networks that can amplify and sustain resistance at both the individual and the systemic level. Only through such comprehensive, coordinated action can we begin to dismantle the structural roots of stigma and create more equitable mental health care environments.

## Data Availability

No datasets were generated or analyzed during the current study.
